# Environmental DNA from Residual Saliva for Efficient Noninvasive Genetic Monitoring of Brown Bears (*Ursus arctos*)

**DOI:** 10.1371/journal.pone.0165259

**Published:** 2016-11-09

**Authors:** Rachel E. Wheat, Jennifer M. Allen, Sophie D. L. Miller, Christopher C. Wilmers, Taal Levi

**Affiliations:** 1 Department of Environmental Studies, Center for Integrated Spatial Research, University of California Santa Cruz, Santa Cruz, California, United States of America; 2 Department of Fisheries and Wildlife, Oregon State University, Corvallis, Oregon, United States of America; 3 Laboratory for Conservation and Utilization of Bio-resource, Yunnan University, Kunming, China; 4 State Key Laboratory of Genetic Resources and Evolution, Kunming Institute of Zoology, Chinese Academy of Sciences, Kunming, China; Senckenberg am Meer Deutsches Zentrum fur Marine Biodiversitatsforschung, GERMANY

## Abstract

Noninvasive genetic sampling is an important tool in wildlife ecology and management, typically relying on hair snaring or scat sampling techniques, but hair snaring is labor and cost intensive, and scats yield relatively low quality DNA. New approaches utilizing environmental DNA (eDNA) may provide supplementary, cost-effective tools for noninvasive genetic sampling. We tested whether eDNA from residual saliva on partially-consumed Pacific salmon (*Oncorhynchus* spp.) carcasses might yield suitable DNA quality for noninvasive monitoring of brown bears (*Ursus arctos*). We compared the efficiency of monitoring brown bear populations using both fecal DNA and salivary eDNA collected from partially-consumed salmon carcasses in Southeast Alaska. We swabbed a range of tissue types from 156 partially-consumed salmon carcasses from a midseason run of lakeshore-spawning sockeye (*O*. *nerka*) and a late season run of stream-spawning chum (*O*. *keta*) salmon in 2014. We also swabbed a total of 272 scats from the same locations. Saliva swabs collected from the braincases of salmon had the best amplification rate, followed by swabs taken from individual bite holes. Saliva collected from salmon carcasses identified unique individuals more quickly and required much less labor to locate than scat samples. Salmon carcass swabbing is a promising method to aid in efficient and affordable monitoring of bear populations, and suggests that the swabbing of food remains or consumed baits from other animals may be an additional cost-effective and valuable tool in the study of the ecology and population biology of many elusive and/or wide-ranging species.

## Introduction

Environmental DNA, or eDNA, has proven to be a comprehensive, noninvasive means of monitoring biodiversity, providing rapid, cost-effective, and efficient insights on species’ distribution and abundance [1,2]. As a tool for applied conservation biology, eDNA is particularly valuable in detection and monitoring of wide-ranging, elusive species [1]. In studies of terrestrial wildlife, hair, scat, and urine are three of the most common sources of DNA for noninvasive monitoring, but eDNA from buccal cells found in saliva can also provide genetic material [3].

eDNA from saliva has been used to identify ungulates [4] and test ungulate resource use [5] and browsing preferences [6], but there have been relatively few studies on the success of obtaining carnivore DNA from saliva, and no studies have yet used eDNA from saliva as a tool for density estimation. Williams *et al*. [7] and Blejwas *et al*. [8] were able to use microsatellites to identify individual coyotes (*Canis latrans*) from saliva collected from bite wounds on sheep. Similarly, Sundqvist *et al*. [9] used saliva collected from bite wounds on sheep to show that domestic dogs (*Canis faimiliaris*) were responsible for attacks. Saito *et al*. [10] identified individual Asiatic black bears (*Ursus thibetanus*) from residual saliva on damaged corn crops. More recently, Farley *et al*. [11] collected brown bear (*Ursus arctos*) saliva from bite wounds on mauling victims and were able to successfully identify individual bears, van Bleijswijk *et al*. [12] identified grey seal (*Halichoerus grypus*) bites on stranded harbour porpoises (*Phocoena phocoena*), and Mumma *et al*. [13] used saliva to help identify predator species at caribou (*Rangifer tarandus*) calf kill sites. Additionally, Harms *et al*. [14] conducted an experimental evaluation of individual identification of captive wolves (*Canis lupus*) and lynx (*Lynx lynx*) using residual saliva from roe deer (*Capreolus capreolus*) carcasses.

Noninvasive research on population genetics and genetic monitoring of individuals has been widely used in the research and management of brown bears, traditionally relying on hair snaring or scat sampling techniques. This includes the use of barbed wire on natural or artificial rub trees [15,16] or arranged around bait stations [17] for hair collection, or collection of fecal samples along transects or roads and game trails [18,19]. Hair collection, however, can be cost- and labor-intensive [20], and the use of hair corrals might not be appropriate for developed areas [21]. Similarly, fecal DNA samples are often degraded in the wet summers of temperate coastal environments, and scats can be difficult to locate in the large, roadless areas that encompass much of the range of brown bears.

Saliva collection provides a potential alternative, or simple augmentation, to hair and scat sampling. In ecosystems throughout the Pacific Northwest, Pacific salmon (*Oncorhynchus* spp.) provide a reliable, discrete, and highly concentrated food resource for brown bears throughout summer and autumn. Salmon are an extremely important resource for brown bears, as bears avoid winter food limitation by storing fat during pulses of spawning salmon and subsequently hibernating during winter. Bears that consume more salmon have been found to have greater body mass, have larger litters, and subsist at higher population densities [22]. Brown bear predation rates on salmon are often high, exceeding 50% in some areas [23], and when run sizes are large, bears may consume as little as 25% of each fish they kill [24]. This high-grading behavior results in the deposition of partially-consumed salmon carcasses, and the residual saliva left on these carcasses when bears abandon them could serve as a source of brown bear DNA for noninvasive population monitoring.

We implemented a project during summer and autumn 2014 in northern Southeast Alaska to determine if saliva collection from partially-consumed Pacific salmon carcasses is a feasible, reliable, effective, and cost efficient source of brown bear DNA. We developed a protocol for saliva sample collection that maximized the likelihood of obtaining individual bear identification from salmon carcasses, and we compared saliva swabbing with swabbing of scat.

## Materials and Methods

### Study area

**Fig 1:**
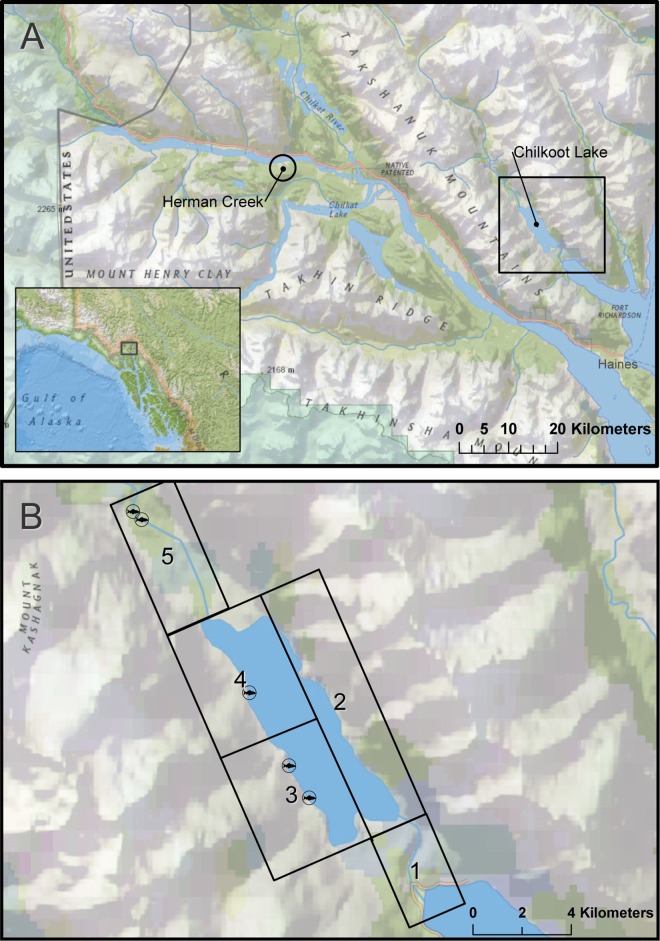
Study Area. (A) Chilkoot and Klehini watersheds near Haines, Alaska, and locations of Chilkoot Lake and Herman Creek where we sampled scat and saliva from sockeye, and saliva from chum carcasses, respectively. (B) We divided the Chilkoot valley into five separate regions, each visited by personnel on a weekly basis. Fish icons mark the locations of known, distinct sockeye salmon spawning areas. Created with ESRI ArcGIS 10.3 software. Basemap data sources include: National Geographic, Esri, DeLorme, HERE, UNEP-WCMC, USGS, NASA, ESA, METI, NRCAN, GEBCO, NOAA, and increment P Corp.

We collected saliva samples from partially-consumed Pacific salmon carcasses at two distinct salmon runs: a midseason run of primarily lakeshore-spawning sockeye salmon (*O*. *nerka*) in the Chilkoot watershed, and a late season run of stream-spawning chum salmon (*O*. *keta*) at Herman Creek in the Klehini watershed, both near Haines, Alaska (Fig 1A). We also swabbed scat samples throughout the Chilkoot watershed, to compare the utility of saliva for monitoring bear populations against this technique. Both study areas are within the boundaries of Haines State Forest. As public domain land, no specific permissions were required for our sampling activities, and our field studies did not involve endangered or protected species.

The Chilkoot watershed is located 12 km NNW of the community of Haines, Alaska. Chilkoot Lake is a glacially turbid lake, approximately 6 km long and 2 km wide. The lower Chilkoot River travels just over 2 km before reaching the ocean. The upper Chilkoot River, originating from the Ferebee Glacier, flows approximately 26 km to the point where it enters Chilkoot Lake. The Chilkoot Valley is narrow, bordered closely to the east by an unnamed mountain range and to the west by the Takshanuk Mountains. A midseason run of sockeye salmon spawns in patches along the western shore of the lake and in the upper river, beginning in early July and extending through early September. Our sampling efforts targeted an abandoned two-track dirt road that parallels the western lake and upper river, and existing game trails that surround the lake, the lower Chilkoot River, and a 6 km reach of the Upper Chilkoot River.

Herman Creek, part of the Klehini watershed, is located approximately 40 km NW of the community of Haines. Two artificial spawning channels along Herman Creek, near where it empties into the Klehini River, serve as part of a wildstock chum salmon enhancement project overseen by the Northern Southeast Regional Aquaculture Association. These channels are currently where the majority of chum salmon returning to Herman Creek spawn. Spawning occurs across several weeks beginning mid-September. We focused our sampling efforts along the banks of both spawning channels.

### Field methods

We collected 272 scat swab samples and 108 saliva samples in the Chilkoot Valley from June through September 2014. We divided the study area into five separate sampling regions each visited once weekly (Fig 1B). Scat swab samples were collected by walking systematically along an established system of roads and game trails in one region per day. We graded scats based on condition, diameter, and estimated age. Visual appearance, presence of mucous and/or insect larvae, and presence of standing water from rainfall on and surrounding scats were used to differentiate between old and fresh scats. Only fresh or relatively fresh scats were selected for sampling. We lightly swabbed the surface of each scat with a single sterile cotton swab, and stored swabs in individual 2 mL microtubes in 100% ethanol [25]. Age classes of bears were determined when possible based on scat diameter (for juveniles) or visual identification of known individuals (for immature bears and adults).

We collected saliva from carcasses by visiting known, distinct spawning grounds (Fig 1B) throughout the study area, and we opportunistically sampled carcasses found elsewhere. Carcasses were swabbed with sterile cotton swabs using a variety of techniques based on the amount and type of tissues consumed: high-graded carcasses, from which only the brain of the fish was eaten, were swabbed along the edges of the braincase; high-graded carcasses with distinct bite holes were swabbed inside of bite holes; carcasses from which strips of skin were removed were swabbed diffusely over exposed muscle tissue or along the torn margins of the skin; fish that had been largely consumed were swabbed along remaining parts, typically the braincase, gill plates, or mandible; some largely intact individuals were diffusely swabbed all along the length of the fish. We kept detailed records of the estimated age of the carcass, swabbing method (e.g. bite marks swabbed individually, diffuse swabbing across exposed muscle tissue, etc.) and the condition of each carcass sampled, and collected photographic evidence of each swabbed carcass. As with scat swab samples, saliva swabs were stored in individual 2 mL microtubes in 100% ethanol. We removed both scat piles and carcasses from the study area following swabbing to avoid repeat sampling. All collected samples were stored at -20°C until extraction.

We collected 80 additional saliva samples for analysis from 48 partially-consumed chum salmon carcasses in the Klehini watershed along Herman Creek in late September 2014. Chum salmon, which spawn at high density in Herman Creek, are more accessible to bears than the lakeshore-spawning sockeye at Chilkoot. Lakeshore spawners are difficult for bears to access [26], as salmon can escape predation by temporarily moving into deeper waters. As bears expend less energy to capture salmon in shallow streams, high-grading behavior is usually more prevalent. Sampling chum salmon at Herman Creek thus allowed us to compare the effectiveness of swabbing salmon in areas with and without high levels of high-grading behavior. We again swabbed carcasses based on consumed tissues. High-grading of chum at Herman Creek also permitted us to swab a single carcass multiple times to compare saliva collection methods within a single sample. For example, both bite holes through the skin and exposed muscle tissue might be present in different areas of one carcass, in which case two different swabs would be taken.

### Genetic methods

Prior to extractions, scat and saliva swabs were removed from ethanol and allowed to dry overnight in a sterile environment. We extracted DNA from all samples using AquaGenomic^TM^ (MultiTarget Pharmaceuticals LLC, Colorado Springs, CO, USA), according to a modified protocol developed from the manufacturer’s instructions. We followed a single extraction protocol for both scat and saliva samples based on the AquaGenomic Stool and Soil Protocol with the following modifications: 1) Swabs were incubated in microfuge tubes with 400μl AquaGenomic for one hour at room temperature to rehydrate; 2) Following rehydration, we added 17.8μl Proteinase K and a small number of 1mm zirconia/silica beads to each microfuge tube; 3) Tubes were then incubated for one hour at 60°C, vortexing every 15 minutes to promote cell disruption, followed by incubation for 15 minutes at 95°C to inactivate the Proteinase K; 4) After pelleting and removal of debris and addition of AquaPrecipi and vortexing, we incubated samples at -20°C for 20 minutes prior to spinning samples down in the centrifuge; and 5) DNA pellets were rinsed three times with 70% ethanol and allowed to air-dry overnight following rinsing.

DNA concentration was not quantified. We avoided contamination by 1) using aerosol resistant pipette tips for critical procedures in DNA extractions and PCR preparations, 2) conducting PCR setup in an appropriate laminar flow PCR cabinet equipped with ultraviolet light and a hepafilter, 3) preparing saliva and fecal samples in a separate location from PCR setups with glove changes between samples, and 4) using negative controls. We screened extracted samples by performing a single duplex PCR to exclude samples from non-target species or of poor quality DNA [27]. We examined duplex PCR products on 1% agarose/GelRed visualized with ultraviolet fluorescence, and kept samples for further analyses if a band from at least one of two loci was visible.

Samples were genotyped at seven microsatellite loci: G1A, G1D, G10B, G10H, G10J, G10M, and G10X [28]. SRY [29] was used for sex determination. We amplified all loci in multiplex PCRs using the Qiagen Multiplex PCR kit (Qiagen, USA). The conditions for PCR are provided in S1 File.

Fluorescence-based detection and sizing of fragments was performed on an ABI 3730 Genetic Analyzer (Applied Biosystems, Foster City, California, USA) at the Oregon State University Center for Genome Research and Biocomputing, and we manually scored alleles using GeneMapper software package, version 4.1 (Applied Biosystems, USA). We required each allele to be detected twice for heterozygotes and an allele to be detected three times for homozygotes to obtain a consensus genotype at each locus. Samples that failed to achieve a consensus for ≥7 loci were dropped from the analysis [27,30]. Genotyping errors, including allelic dropout and scoring errors, were estimated by comparing repeated genotypes (recaptures) to consensus genotypes using ‘gimlet’ [31].

We identified individual genotypes using the R package ‘allelematch’ [32]. The package uses maximum likelihood estimates with the Hamming distance method [33] to find similarities between samples, and uses dynamic hierarchical clustering with the Dynamic Tree Cut package for R [34].

Population estimates for samples collected at Chilkoot were generated with the single-session approach of the R package ‘capwire’ [35], developed specifically for noninvasive genetic samples [36]. Data from both saliva and scat collection were overdispersed. We used the PART algorithm in capwire to partition the data and fit a two innate rates model (max. population 80) to count data in the lower partition, then added individuals from the upper partition to the maximum likelihood estimate of population size *post hoc*. We performed a parametric bootstrap (1,000 bootstraps) to obtain confidence intervals around our population size estimates [35].

We examined the effectiveness of saliva swab versus scat swab sampling techniques using the following criteria: genotyping success (the proportion of scats or salmon carcasses, respectively, from which we were able to successfully amplify brown bear DNA and achieve a consensus genotype at ≥7 loci), genotyping error rate, number of bears identified, and size of the population estimate and confidence intervals. Pairwise differences in the genotyping success rates of saliva versus scat samples were examined by using the *z* test for proportions. The *z* test for proportions was also used to compare the proportional genotyping success rates of each saliva swabbing technique. We generated population size estimates and confidence intervals for scat and saliva samples separately and combined. Additionally, we assessed the efficiency of sampling methods by comparing search effort and costs needed for saliva versus scat swab sampling. Cost per sample, cost per genotyped sample, and cost per bear identified were calculated by summing estimates of field labor costs required to collect each sample, lab labor costs for processing each sample, and costs for DNA extraction, multiplex PCR, and genotyping for each sample, excluding consumables.

## Results

We sampled a total of 272 scats and 156 partially-consumed salmon carcasses between June and September 2014 (Table 1). Of these, 42.3% of scat swab samples and 13.8% of saliva swab samples failed to produce amplifiable product. Of the remaining samples, 189 were successfully amplified at 7–8 loci (including the SRY sex locus). These 97 saliva and 92 scat swab samples were assigned to individuals. Variability of the selected microsatellite loci was high, with *H*_*o*_ = 0.74 and three to seven alleles per locus. *P*_ID_ (1.41e^-6^) and *P*_ID(sib)_ (3.14e^-3^) were low; the *P*_ID(sib)_ threshold for accepting a genotype was 0.05. All PCR replicates of the sex marker SRY gave consistent results. We identified 62 individuals: 44 female and 18 male.

**Table 1:** Results by Sample Type.

Sample Type	Samples Collected	Successfully Genotyped	Unique Bears Identified
**Scat**	272	92	29
**Saliva**	188	97	44
**-Sockeye**	108	49	23
**-Chum**	80	48	22
**Combined**	460	189	62

### Evaluation of swabbing techniques for carcasses

**Fig 2:**
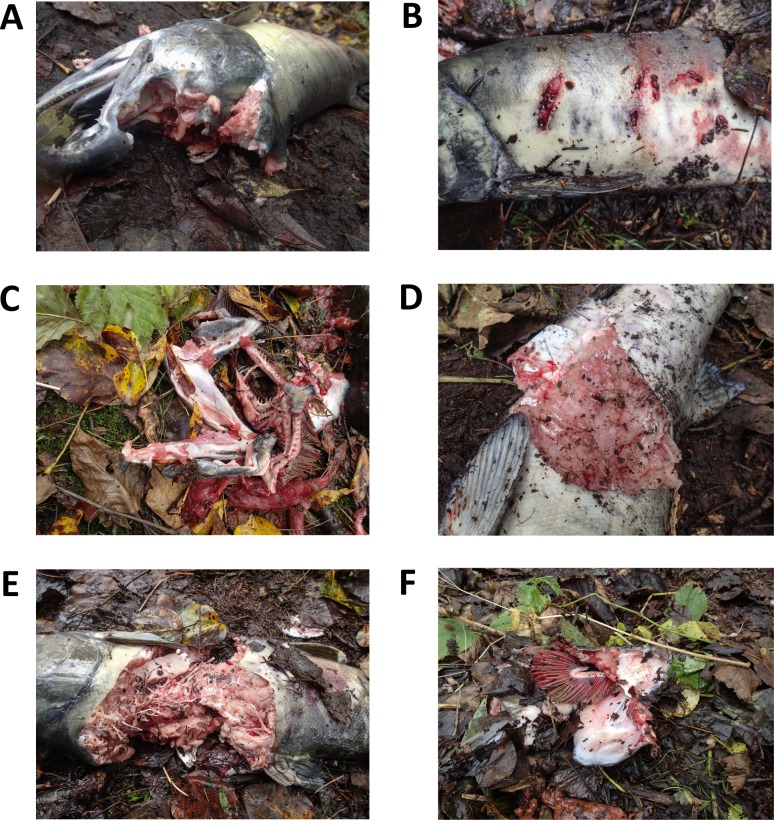
Salmon Carcass Categorization. We categorized carcasses based on the type of consumption observed: (A) braincase; (B) bite holes; (C) mandible; (D) margins of torn skin; (E) exposed muscle tissue; (F) gill plates. A wide range of carcass types produced bear DNA that amplified successfully; saliva samples from each of the carcasses pictured were successfully genotyped to individual.

**Fig 3:**
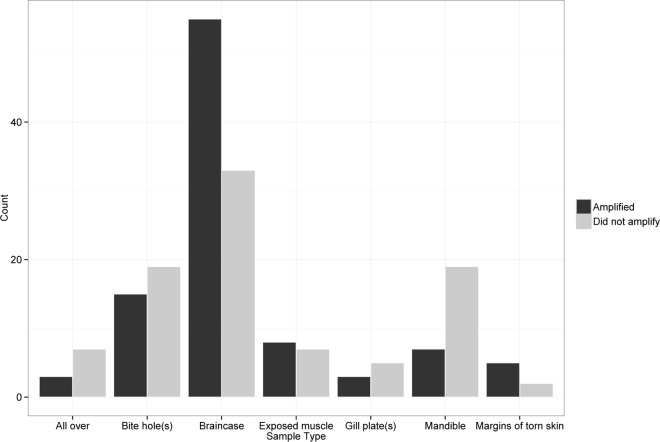
Relative Success of Different Salmon Swabbing Techniques. Braincases and bite holes were encountered most frequently, and also amplified at proportionally higher rates than other swabbing techniques.

We generally found that a wide range of carcass types produced usable DNA. Swabbing technique was broken up into eight categories based on unconsumed tissues (Fig 2, Table 2). Braincases, from both high-graded and mostly consumed fish, were the most common areas swabbed (N = 88), followed by bite holes (N = 34). Margins of torn skin had the best amplification rate, at 71%, compared to other swabbing techniques (Table 2), but these types of carcasses were encountered infrequently (N = 7). We achieved consensus genotypes at ≥7 loci from 62% of braincase swabs, the highest rate of successful amplification, followed by bite holes at 44%. Amplification success rates were lower for swabs taken from gill plates (38%), mandibles (27%), exposed muscle (21%), and swabs swiped diffusely all over the surface of the length of the fish (30%). Amplification success for braincase swabs was significantly higher than all other swabbing techniques (*z* = 3.00, *P* < 0.01; Fig 3).

**Table 2:** Saliva Swabbing Techniques and Results. “Proportion amplified” refers to the proportion of samples from which we were able to successfully amplify brown bear DNA and achieve a consensus genotype at ≥7 loci.

Area Swabbed	Description	No. Sampled	Proportion amplified
Braincase	A high-graded salmon where only the brain and eyes of the fish have been removed, or a largely consumed carcass where only the braincase and possibly the mandible of the salmon remain. Swab rolled along the edges of the braincase.	88	0.62
Bite hole(s)	A high-graded salmon where distinct bite hole(s) are present penetrating the skin of the fish. Swab inserted into cavity made by tooth.	34	0.44
Mandible	Salmon almost entirely consumed; only one or both halves of the mandible remain. Swab rolled along both interior and exterior of mandible.	26	0.27
Exposed muscle	A high-graded salmon where a portion of skin has been removed and some muscle tissue has been consumed. Swab wiped diffusely over exposed muscle, targeting areas where consumption has occurred.	15	0.21
All over	A carcass that is largely untouched or a high-graded salmon where only the brain and eyes have been removed. Swab swiped diffusely over the surface of the skin along the entire length of the fish.	10	0.30
Gill plate(s)	Salmon almost entirely consumed; only one or both gill plates remain. Swab rolled along both interior and exterior of gill plate(s)	8	0.38
Margins of torn skin	A high-graded salmon where a portion of skin has been removed and muscle tissue appears largely untouched. Swab rolled along margins of torn skin, targeting areas where bite likely originated.	7	0.71

High-grading was far more prevalent at Herman Creek than at Chilkoot, which allowed us to take multiple swabs from single carcasses. Out of 48 carcasses sampled at Herman Creek, we collected between two and four swabs from 21 fish. Bite holes were the most common swabbing location (N = 22), followed by braincases (N = 19) and exposed muscle (N = 7); in most cases the braincase of the carcass was swabbed in addition to one or more bite holes. Of carcasses swabbed in multiple locations, braincase samples were most likely to successfully amplify—57.9% of braincase swabs from carcasses sampled multiple times were assigned to individual with a consensus genotype at ≥7 loci, compared to 31.8% of swabs of bite hole(s) and 28.6% of swabs of exposed muscle tissue.

### Evaluation of scat versus saliva sampling

#### Effectiveness: Genotyping success and population estimation

Consensus brown bear genotypes at ≥7 loci were achieved for 55% of all salmon carcasses sampled for saliva. Genotyping success was highest (77%) at Herman Creek, where high-grading behavior was prevalent. At Chilkoot, where high-grading behavior was uncommon and salmon carcasses were sparse, the genotyping success rate was 45%. The genotyping success rate for saliva collected from high-graded carcasses at Herman Creek was significantly higher than for saliva collected at Chilkoot (*z* = 1.98, *P* < 0.05).

Consensus brown bear genotypes at ≥7 loci were achieved for 34% of all sampled scats. Genotyping success was significantly higher for saliva than for scat swab samples (*z* = 5.69, *P* < 0.01). Additionally, our calculated genotyping error rate was slightly lower for saliva than for scat swab samples. Per-locus error rates for scat swab samples averaged 10.6% across all loci for scat samples, while the error rates for saliva averaged 8.2%.

**Fig 4:**
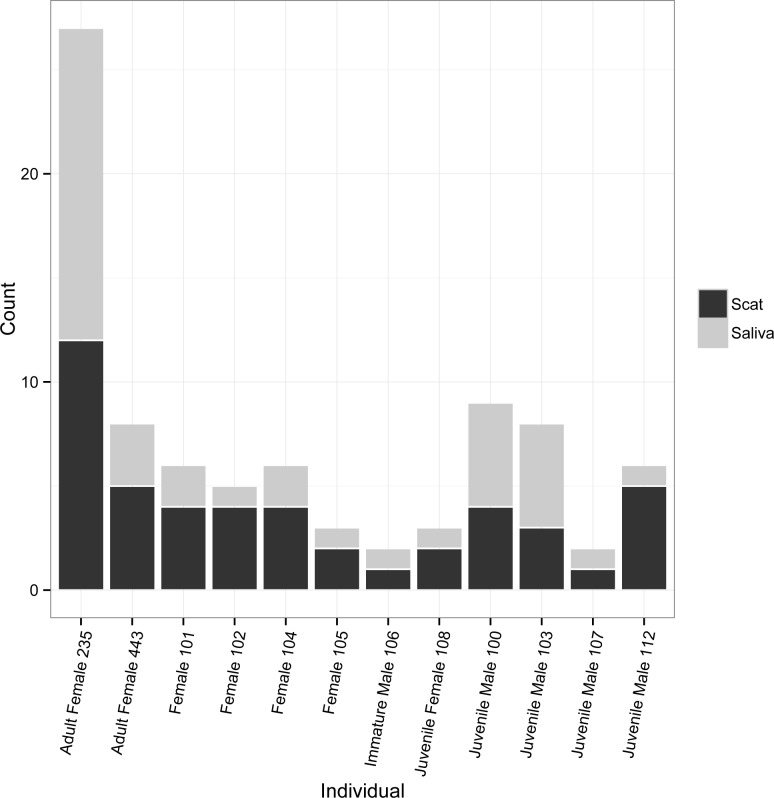
Detections by Sample Type. Number of detections by sample type for 12 individual brown bears identified from both saliva samples and scat swab samples collected in the Chilkoot valley near Haines, AK. Age classes, where noted, were determined based on scat diameter (for juveniles) or sightings of known individuals (for immature bears and adults).

Neither scat nor saliva samples collected at Chilkoot detected all bears, but 29 out of 40 individuals were identified from 92 of 272 scat swab samples that successfully genotyped, and 23 individuals were detected using saliva from 49 of 108 sockeye salmon carcasses that successfully genotyped. Twelve bears were detected with both saliva and scat swabbing (Fig 4). Individuals were detected from 1 to 26 times throughout the sampling season, with an average of 2.0 saliva samples per bear and 3.2 scat swab samples per bear. Combining scat and saliva detections increased detection frequencies slightly, with individual bears sampled an average of 3.4 different times.

Population size estimates for Chilkoot varied based on sample type (Table 3). The population size estimate for saliva samples alone was the lowest at 29 individual bears, with 95% confidence intervals of 24–36. The population size estimate generated using scat swabs alone was 39, with slightly wider confidence intervals (31–48) than saliva alone. Combining scat and saliva samples slightly increased the number of detections per bear, resulting in a population estimate of 42 bears and reducing confidence intervals to 41–43. In all cases, these estimates are likely affected by an upward bias derived from violation of the assumption of a closed population.

**Table 3:** Population Estimates for Chilkoot Valley by Sample Type.

Sample Type	No. Samples	Population Size Estimate	95% Confidence Intervals
**Scat**	92	39	31–48
**Saliva**	49	29	24–36
**Combined**	141	42	41–43

#### Efficiency: Cost and labor

**Fig 5:**
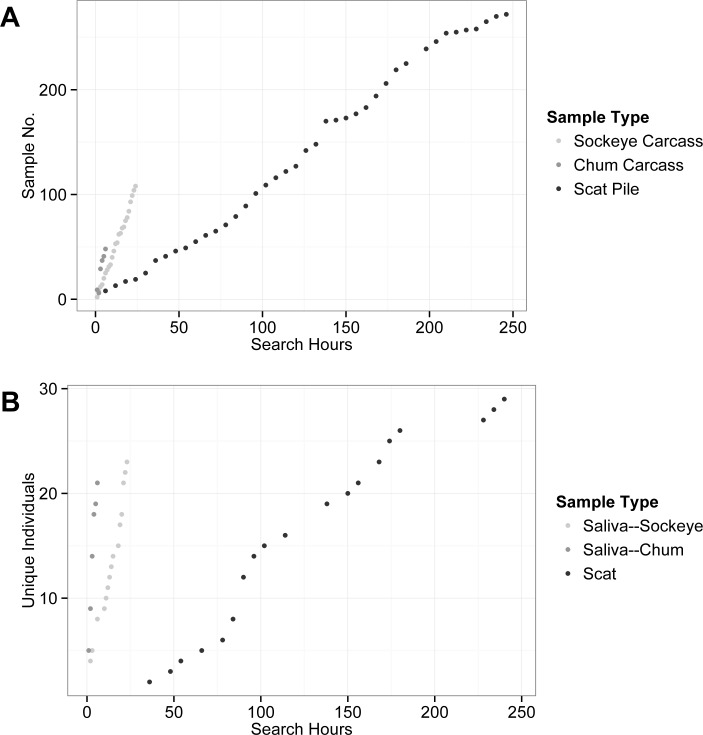
Search Effort. Number of search hours required to locate new samples (A) and unique individual bears (B) based on sample type. Saliva samples from sockeye salmon and scat samples were collected from June through early September in the Chilkoot Valley, while saliva samples from chum salmon carcasses were collected in late September at Herman Creek.

Cost per sample, cost per genotype, and cost per bear identified were higher for scat swabs than saliva (Table 4). Costs per sample averaged $39 for scat swab versus $28 for saliva. Cost per genotype averaged $117 for scat swabs and $55 for saliva. Cost per bear averaged $370 needed to identify each individual using scat swabs versus $118 needed to identify each bear using saliva. In general, laboratory expenses for processing and genotyping samples were similar for scat swab and saliva samples, but field labor costs were substantially higher for sampling scat, at an average of $18 per sample, than saliva, at an average of just over $4 per sample. This discrepancy stems from the number of search hours required to locate salmon carcasses versus scats. Many more search hours were required per sample and per individual bear for scat than for saliva (Fig 5). On average, only 0.22 search hours were required to locate each sockeye salmon carcass, and 0.13 search hours required to locate each chum salmon carcass, compared to an average of 0.90 hours required to locate each scat. Additionally, it took an average of 8.5 hours of searching for and sampling scat at Chilkoot to identify each individual bear, a much higher rate than that for sampling saliva from sockeye carcasses, at 1.04 search hours/unique bear. Saliva samples obtained from high-graded carcasses required the lowest search effort overall—on average it took just 0.27 hours of search time at Herman Creek to identify each bear.

**Table 4:** Cost (USD) by sample type.

Sample Type	Cost per sample	Cost per genotype	Cost per unique bear
**Scat**	39	117	370
**Saliva**	28	55	118
**Overall**	35	85	217

## Discussion

Our findings support the application of eDNA from residual saliva as a low-effort, cost-effective tool to aid in the monitoring of brown bear populations. Residual saliva collected from partially-consumed salmon carcasses provided large sample quantities in discrete geographic locations, detected multiple individual bears of both sexes and multiple age classes, and successfully detected individuals on multiple occasions (recaptures). Combining scat and saliva samples collected at Chilkoot Lake slightly increased the number of detections per bear and decreased the confidence intervals associated with our population estimate. When collected opportunistically during the course of hair snaring or scat collection projects, saliva samples from salmon carcasses could increase the number of detections and the number of bears identified with very little additional effort.

In regions where black bears (*Ursus americanus*) consume salmon, saliva sampling should be suitable for simultaneously monitoring both species because black bears share many of the same microsatellite loci with brown bears. Some of these shared loci (e.g. G10J) have discrete alleles for each species, thus allowing for species differentiation [27]. Although brown and black bears are sympatric in Haines, Alaska, we did not find DNA from any black bears, which is consistent with previous research suggesting that black bears are locally excluded from salmon in this system by brown bears [37].

The reduced labor and cost associated with monitoring bear populations by sampling saliva from salmon carcasses could prove particularly advantageous in locales where infrastructure is lacking. In remote or protected areas where vehicular traffic is restricted, such as most of Southeast Alaska and coastal British Columbia, salmon swabbing could supplement hair snaring or scat collection, as many spawning areas are easily accessible by boat. We envision this method being particularly useful in ecosystems with large runs of stream-spawning salmon such as chum and pink (*O*. *gorbuscha*) salmon, where high-grading behavior by bears is prevalent.

Many animals are faced with boom-bust cycles of food availability. In response to this, many eat only the choicest portion and drop the remainder (monkeys and other arboreal mammals, birds, and some lizards and some marine mammals), some cache food (rodents, felids, bears, foxes, large lizards), and many fight over large carcasses between themselves and other species, which often results in leaving bits of a carcass behind. In all of these cases, they also leave saliva behind in a convenient manner for researchers. It is this response to a boom and bust food resources that allowed us to efficiently collect brown bear saliva. As an additional resource for sampling individuals who are often widely dispersed or hard to follow, saliva sampling is invaluable. Further, this type of saliva sampling can also provide behavioral and ecological information when each sample is georeferenced.

### Evaluation of scat versus saliva sampling

Genotyping success rates were higher for saliva than for scat swab samples. Genotyping success rates for scat swab samples averaged only 34%. Although other microsatellite studies utilizing brown bear scats have achieved success rates as high as 80% [38,39], our genotyping success rate using scat swabs is well within the range of success rates achieved by other studies (S1 Table). It is possible, however, that alternative sampling, collection, and/or storage techniques for scats would improve success and decrease the discrepancy between our genotyping success rate for scat swabs and saliva.

In addition to greater genotyping success rates, search hours necessary to locate carcasses were much lower for saliva than for scat swab samples, with the effect of greatly reducing labor, and subsequently costs, for saliva sample collection compared to scat. Search effort was lowest overall, and genotyping success rate highest, at Herman Creek, where dense aggregates of spawning salmon in a relatively shallow streambed led to high predation rates by bears and frequent high-grading behavior. At Herman Creek, we collected samples identifying 22 bears in four sampling bouts that spanned fewer than six hours total. Regular, brief visits to spawning areas, particularly during salmon runs when high-grading of carcasses is prevalent, could effectively sample a substantial proportion of the local bear population with very little effort.

When compared to scat swab sampling, saliva samples from Chilkoot detected fewer bears, and population size estimates generated using scat swabs alone were higher than estimations generated with saliva alone. However, we spent more than 200 additional hours searching for scat than for salmon for saliva collection, and we collected more than one and a half times more scat swabs than saliva samples at Chilkoot. Although the sockeye salmon run at Chilkoot Lake in 2014 surpassed the 10-year average for the region [40], lakeshore spawners are difficult for bears to access [26], resulting in fewer high-graded carcasses. Taken together, these results suggest that, as a supplementary sampling method to traditional hair or scat sampling, saliva sampling represents a low-effort tool to identify and monitor individuals.

### Evaluation of swabbing methods for carcasses

Braincase removal was by far the most common consumption type we encountered; even in cases where the majority of the salmon was consumed, bears would often leave the de-brained skull and gill plates of the fish. Further, braincase swabs had the highest proportional success rate in terms of amplification and assignment to individual. Individual bite holes were also frequently encountered in abandoned carcasses, particularly in high-graded carcasses, and had an only slightly lower amplification success rate than braincase swabs.

Although braincase and bite hole swabs were generally the most useful, we found a wide range of carcass types and conditions to successfully amplify bear DNA (Fig 2). While we recommend that those looking to adopt this technique in their own work target braincases and individual bite holes when swabbing for saliva, we suggest that researchers keep in mind the relative low costs of collection, DNA extraction, and PCR screening for DNA quality—the margins of any area of a salmon that has been consumed are potential reservoirs for usable bear DNA. Additionally, while we were able to successfully obtain and amplify DNA using dry sterile cotton swabs rolled or swiped along the surface of a carcass, we encourage exploration of alternative collection protocols. For example, swabbing surfaces very lightly or spraying swabs with deionizied water prior to swabbing might increase amplification success.

### Limitations

Our results demonstrate that residual saliva collected from partially-consumed salmon carcasses is a feasible noninvasive genetic sampling tool to supplement traditional hair and/or scat sampling. We believe this technique could be implemented broadly throughout both the Pacific Northwest and coastal Asia in areas where bears consistently feed on spawning salmon. As this is the first time partially-consumed salmon carcasses have been used to sample bear DNA and the first time salivary DNA has been used for population estimates, we recognize that this method may have limitations. First, it is possible that strictly sampling saliva could bias population estimates, as individual bears may have different levels of fishing success or consumption behaviors that could contribute to capture heterogeneity and violation of assumptions for mark-recapture models. Our saliva sampling identified bears from both sexes and multiple age classes, but neither saliva sampling nor scat sampling identified all individuals, and confidence intervals were lowest for population estimates that included both scat and saliva samples. Several approaches have been proposed in mark–recapture modeling for dealing with capture heterogeneity—for example, capwire’s ‘two innate rates model’ mixes relative capture probability of harder to capture individuals with individuals that are easier to capture [36]—and we suggest researchers looking to implement saliva sampling in their own studies take into account potential violations of capture homogeneity assumptions.

Second, although genotyping success rates for saliva samples averaged 55%, this number is lower than some bear population studies using hair snares [21,41,42], as well as some studies using scat [38,39,43]. Research has illustrated that amplification rates of noninvasive samples decrease and genotyping error rates increase with increasing precipitation [43,44]. Our genotyping success rates were probably negatively influence by an extremely wet summer. The summer of 2014 was the wettest summer in nearly a century in Haines, with record-breaking rainfall nearly every month of the study. Given the difficult field conditions and intense precipitation, we expect greater amplification success could be expected for both scat and saliva samples in drier years. Additionally, while we found saliva sampling to have greater genotyping success rates than scat, it is possible that alternative sampling, collection, and/or storage techniques for scat samples might reverse this relationship. Although we selected scats in the field that were graded as fresh or relatively fresh, grading criteria are subjective and might not accurately identify the true age of a scat. Stricter criteria for sampling scats (e.g., not collecting any scat following rainfall, only swabbing scats with visible mucous present) may improve genotyping success rates and decrease costs for scat swabs.

Third, swabs of partially-consumed salmon carcasses also sample salmon DNA, sometimes in large quantities, in addition to bear DNA. We had to discount two microsatellites (G10C and Mu59) when testing loci to use for our study as a result of apparent amplification of salmon DNA within the region of the bear marker. Scat swab samples tested using these markers amplified normally, and although some saliva samples produced identifiable alleles, the majority were consistently obscured. Murphy *et al*. [45] found fecal samples from captive brown bears fed diets of Atlantic salmon (*Salmo* spp.) had very low amplification success rates, and suggested this might be a result of interference with salmonid by-products, which could explain both the issues we encountered with these markers and the low amplification success rates of scat swab samples. Limitations with these markers could be overcome by swabbing carcasses less intensively (i.e. lessening the quantity of salmon tissue picked up on the swab), by additional optimization of DNA extraction, or PCR amplification methods, such as the use of salmon-blocking oligonucleotides.

Additionally, because of the nature of bear feeding behavior, it is possible for carcasses to become contaminated with DNA from a second individual bear if scavenging occurs following the initial consumption event. In populations with high allelic diversity and heterozygosity, however, mixed samples should be detectable [46]. Contamination could also occur as a result of scavenging behavior from other species (e.g. coyote, mink [*Neovison vison*], marten [*Martes americana*], etc.), particularly if the microsatellites used are shared among species. For example, the sex marker we used, SRY, amplifies in multiple mammalian species. Where contamination is possible, researchers should consider utilizing species-specific markers. Studies utilizing markers that overlap among species should ensure that all PCR replicates give consistent results. These considerations aside, saliva is a promising new source for bear DNA in salmon ecosystems.

### Utility of eDNA from residual saliva for population monitoring

We found residual saliva collected from partially-consumed salmon carcasses to be a successful, labor- and cost-effective way to sample brown bear populations. As far as we are aware, this study is the first to use noninvasively collected saliva to estimate the population density of any species of wildlife. As we focused primarily on the methodology of saliva sampling in this study, we recommend that researchers looking to implement this technique as a method to monitor bear populations take into account potential capture heterogeneity and violation of assumptions for mark-recapture models that may arise due to differing levels of fishing success or consumption behaviors among individual bears.

Although salmon carcasses provide a readily available source of saliva to aid in the monitoring of bear populations, baited saliva sampling should be easily generalizable to systematic surveys of carnivores if appropriate baits can be identified and tested [47]. As an eDNA source, salivary DNA can complement, or perhaps in some conditions replace, alternative sampling methods, particularly when these methods are inefficient, prohibitively expensive, or difficult to implement. For example, some elusive small carnivore species might be more easily tempted to deposit saliva on bait rather than enter a hair collection tube or trap. Large canids and felids are particularly difficult to survey when scats are not available because researchers typically rely on scented and barbed pads or boards to elicit rubbing behavior in the hope of obtaining hair samples for genetic monitoring. Rub boards could be easily paired with durable baits that might prompt an animal to deposit saliva even when that animal is not provoked to rub on a board or pad. eDNA from saliva for noninvasive genetic monitoring has the potential to be an important tool to supplement existing approaches for the conservation and management of wildlife populations.

## Supporting Information

S1 Table Genotype Success Rates from Previous *U*. *arctos* Studies Utilizing Scat Collection. (DOCX)

S2 Table Genotype Success Rates from Previous Studies Utilizing Saliva Collection. (DOCX)

S1 Fig Number of Samples that Successfully Genotyped Versus Number of Unique Individuals Identified Across All Samples Collected. Number of samples that successfully genotyped (grey squares—scat, grey diamonds—saliva) versus number of unique individuals identified (red squares—scat, blue diamonds—saliva) across all samples collected, with trendlines.
(TIF)

S1 File Protocols for microsatellite PCR and data analyses used for genotyping noninvasive saliva and scat samples collected for brown bears in Southeast Alaska. (DOCX)
