# Supplementary material for: Environmental DNA from Residual Saliva for Efficient Noninvasive Genetic Monitoring of Brown Bears (Ursus arctos)
Source: PLoS One. 2016 Nov 9;11(11):e0165259. doi: 10.1371/journal.pone.0165259 (PMC5102439; doi:10.1371/journal.pone.0165259)
Supplement: S1 Table — (DOCX) [file pone.0165259.s001.docx]

**S1 Table**

| **Study** | **Fecal sampling method** | **Genotyping success rate** |
| --- | --- | --- |
| Taberlet *et al.* 1997 | Qty not reported, samples dried | 20% |
| Murphy *et al.* 2003 | Collected whole, freeze-dried, and ground | 76% |
| Bellemain *et al.* 2005 | 1 cm^3^ of scat in a 20-mL bottle with 95% ethanol | 70-80% |
| Murphy *et al.* 2007 | 6 g of mixed faeces were collected on silica in an air-tight vial | 53% |
| Pérez *et al.* 2008 | Qty not reported, samples soaked in ethanol then dry stored in silica | 43.5% |
| Skrbinšek *et al.* 2010 | Qty not reported, samples stored in 50 mL screw-cap tubes with 96% ethanol | 88.2% |
| De Barba *et al.* 2010 | 10 mL of homogenized faeces stored in 40 mL of 95% ethanol | 22% |
| Stenglein *et al.* 2010 | 10 mL sample preserved in 40 mL of 95% ethanol | 48-59% |
| Kruckenhauser *et al.* 2008 | 1 cm^3^ of scat material stored in 96% ethanol | 35% |
| Kindberg *et al.* 2011 | 1 cm^3^ of the sample into a 20-ml collection bottle | 73% |
| Straka *et al.* 2011 | Qty not reported, samples stored in stored in 96% ethanol | 57% |

**References**

Bellemain, E., J. E. Swenson, D. Tallmon, S. Brunberg, and P. Taberlet. 2005. Estimating Population Size of Elusive Animals with DNA from Hunter-Collected Feces: Four Methods for Brown Bears. Conservation Biology 19:150–161.

De Barba, M., L. P. Waits, P. Genovesi, E. Randi, R. Chirichella, and E. Cetto. 2010. Comparing opportunistic and systematic sampling methods for non-invasive genetic monitoring of a small translocated brown bear population. Journal of Applied Ecology 47:172–181.

Kindberg, J., J. E. Swenson, G. Ericsson, E. Bellemain, C. Miquel, and P. Taberlet. 2011. Estimating population size and trends of the Swedish brown bear Ursus arctos population. Wildlife Biology 17:114–123.

Kruckenhauser, L., G. Rauer, B. Däubl, and E. Haring. 2008. Genetic monitoring of a founder population of brown bears (Ursus arctos) in central Austria. Conservation Genetics 10:1223–1233.

Murphy, M. A., K. C. Kendall, A. Robinson, and L. P. Waits. 2007. The impact of time and field conditions on brown bear ( Ursus arctos ) faecal DNA amplification - Springer. Conservation Genetics 8:1219–1224.

Murphy, M. A., L. P. Waits, and K. C. Kendall. 2003. The influence of diet on faecal DNA amplification and sex identification in brown bears (Ursus arctos). Molecular Ecology 12:2261–2265.

Pérez, T., F. Vázquez, J. Naves, A. Fernández, A. Corao, J. Albornoz, and A. Domínguez. 2008. Non-invasive genetic study of the endangered Cantabrian brown bear (Ursus arctos). Conservation Genetics 10:291–301.

Skrbinšek, T., M. Jelenčič, L. Waits, I. Kos, and P. Trontelj. 2010. Highly efficient multiplex PCR of noninvasive DNA does not require pre-amplification. Molecular Ecology Resources 10:495–501.

Stenglein, J. L., M. De Barba, D. E. Ausband, and L. P. Waits. 2010. Impacts of sampling location within a faeces on DNA quality in two carnivore species. Molecular Ecology Resources 10:109–114.

Straka, M., L. Paule, O. Ionescu, J. Štofík, and M. Adamec. 2011. Microsatellite diversity and structure of Carpathian brown bears (Ursus arctos): consequences of human caused fragmentation. Conservation Genetics 13:153–164.

Taberlet, P., J.-J. Camarra, S. Griffin, E. Uhrès, O. Hanotte, L. P. Waits, C. Dubois-Paganon, T. Burke, and J. Bouvet. 1997. Noninvasive genetic tracking of the endangered Pyrenean brown bear population. Molecular Ecology 6:869–876.
