# Supplementary material for: Environmental DNA from Residual Saliva for Efficient Noninvasive Genetic Monitoring of Brown Bears (Ursus arctos)
Source: PLoS One. 2016 Nov 9;11(11):e0165259. doi: 10.1371/journal.pone.0165259 (PMC5102439; doi:10.1371/journal.pone.0165259)
Supplement: S2 Table — (DOCX) [file pone.0165259.s002.docx]

**S2 Table**

| **Study** | **Type** | **Genotyping success rate** |
| --- | --- | --- |
| Williams *et al.* 2003 | Species identification | 95% |
| Blejwas et al. 2006 | Individual identification | 58% |
| Saito *et al.* 2008 | Individual identification | 30% |
| Nichols *et al.* 2012 | Species identification | 12.5 - 100% |
| Mumma *et al.* 2014 | Species Identification | 46 - 86% |
| Nichols *et al.* 2015 | Species identification | 54% |
| Nichols *et al.* 2015b | Species identification | 31% |
| Harms *et al.* 2015 | Individual identification | < 50 to >83% |

**References**

Blejwas, K. M., C. L. Williams, G. T. Shin, D. R. McCullough, and M. M. Jaeger. 2006. Salivary DNA Evidence Convicts Breeding Male Coyotes of Killing Sheep. Journal of Wildlife Management 70:1087–1093.

Harms, V., C. Nowak, S. Carl, and V. Muñoz-Fuentes. 2015. Experimental evaluation of genetic predator identification from saliva traces on wildlife kills. Journal of Mammalogy 96:138–143.

Mumma, M. A., C. E. Soulliere, S. P. Mahoney, and L. P. Waits. 2014. Enhanced understanding of predator–prey relationships using molecular methods to identify predator species, individual and sex. Molecular Ecology Resources 14:100–108.

Nichols, R. V., J. P. G. M. Cromsigt, and G. Spong. 2015a. DNA left on browsed twigs uncovers bite-scale resource use patterns in European ungulates. Oecologia 178:275–284.

Nichols, R. V., J. P. G. M. Cromsigt, and G. Spong. 2015b. Using eDNA to experimentally test ungulate browsing preferences. SpringerPlus 4:1–5.

Nichols, R. V., H. Königsson, K. Danell, and G. Spong. 2012. Browsed twig environmental DNA: diagnostic PCR to identify ungulate species. Molecular Ecology Resources 12:983–989.

Saito, M., K. Yamauchi, and T. Aoi. 2008. Individual Identification of Asiatic Black Bears Using Extracted DNA from Damaged Crops. Ursus 19:162–167.

Williams, C. L., K. Blejwas, J. J. Johnston, and M. M. Jaeger. 2003. A Coyote in Sheep’s Clothing: Predator Identification from Saliva. Wildlife Society Bulletin 31:926–932.
