# Supplementary figures and images for: Environmental DNA from Residual Saliva for Efficient Noninvasive Genetic Monitoring of Brown Bears (Ursus arctos)
Source: PLoS One. 2016 Nov 9;11(11):e0165259. doi: 10.1371/journal.pone.0165259 (PMC5102439; doi:10.1371/journal.pone.0165259)

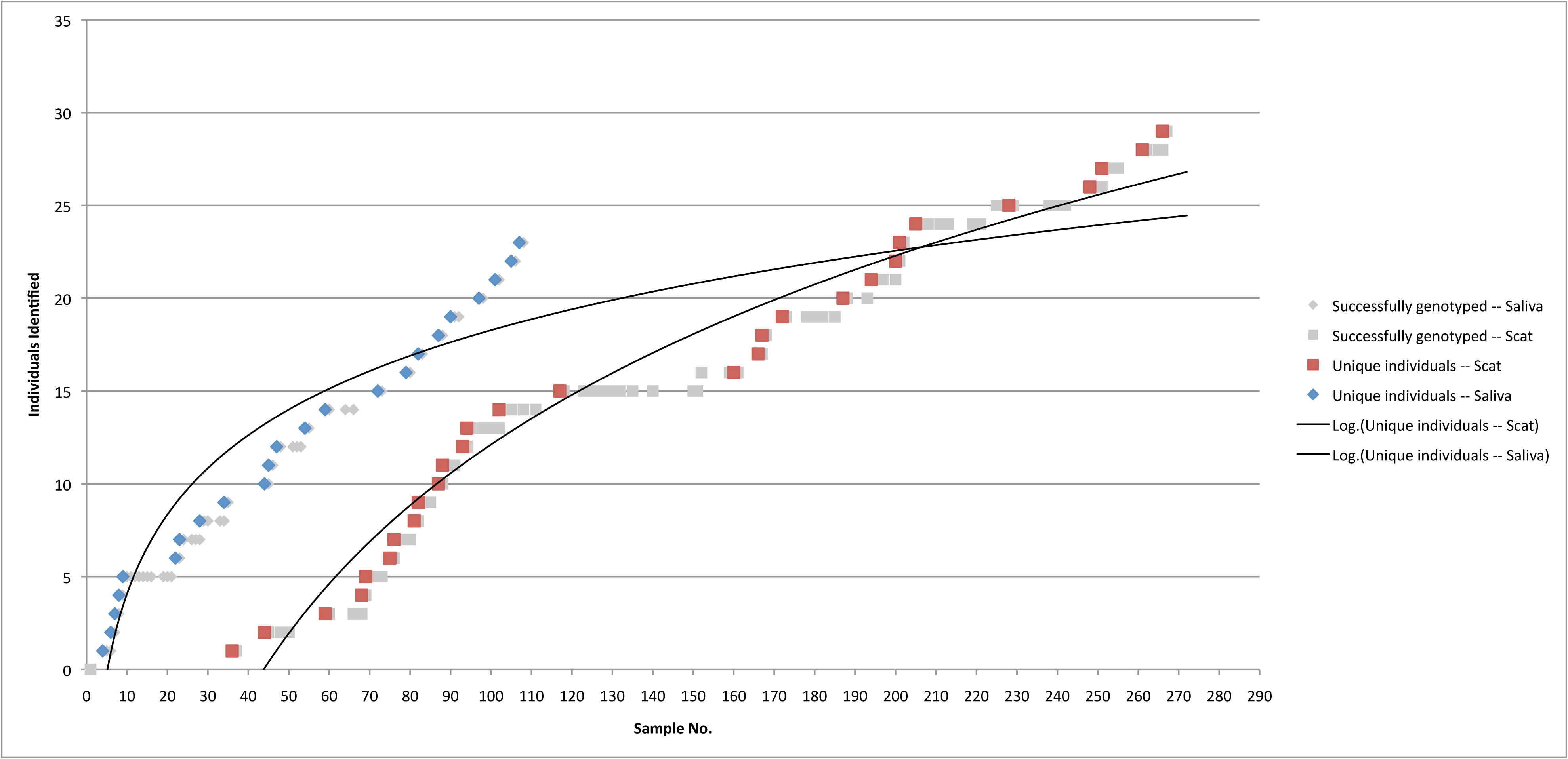

Supplement: S1 Fig — Number of samples that successfully genotyped (grey squares—scat, grey diamonds—saliva) versus number of unique individuals identified (red squares—scat, blue diamonds—saliva) across all samples collected, with trendlines. (TIF) [file pone.0165259.s003.tif]
