# Supplementary material for: Environmental DNA from Residual Saliva for Efficient Noninvasive Genetic Monitoring of Brown Bears (Ursus arctos)
Source: PLoS One. 2016 Nov 9;11(11):e0165259. doi: 10.1371/journal.pone.0165259 (PMC5102439; doi:10.1371/journal.pone.0165259)
Supplement: S1 File — (DOCX) [file pone.0165259.s004.docx]

**Protocols for microsatellite PCR and data analyses used for genotyping noninvasive saliva and scat samples collected for brown bears in Southeast Alaska.**

*Microsatellite PCR*

To genotype samples, we separated primers into two groups (Table 1) for multiplex PCR. Multiplex PCRs were performed using Qiagen Multiplex PCR kits in 12μl volume, with reactions containing 2X Qiagen master mix, 50mg/ml BSA, 0.1-0.2 mM of each primer, 2μl template DNA, and Milli-Q water to 12μl. Reactions for multiplex group 2 (Table 1) also contained 5X Qiagen Q-solution. The PCR profile was 15 min at 95°C initial denaturation followed by 39 cycles of 30 s for denaturation at 94°C, 90 s for annealing at 56°C, and 60 s for extensions at 72°C, followed by a final extension step of 30 min at 60°C. PCR products were loaded on an ABI 3730 Genetic Analyzer and scored using GeneMapper 4.1.

Samples were first multiplexed for group 1 and were discarded if they did not produce scorable results at ≥ 2 loci. Samples multiplexed for both groups were amplified a minimum of three times to ensure reliable results. Individuals missing two or fewer loci (e.g. failed PCR, poor-quality DNA extract) from both groups were amplified an additional three times; samples missing more than two loci were excluded from our data set.

*Protocol for genetic analyses*

We identified unique genotypes using the R package ‘allelematch’ (Galpern et al. 2012), using a mismatch parameter of four alleles. We used program ‘gimlet’ (v 1.3.3, Valière 2002) to estimate heterozygosity, number of alleles per locus, and calculate *P*_ID_ and *P*_ID(sib)_. Genotyping error was estimated by comparing repeated genotypes (recaptures) to consensus genotypes using gimlet.

**Table 1:** Sequences (5' - 3') of the microsatellite primers used for genotyping scat and saliva samples collected noninvasively for the brown bear in Southeast Alaska. Loci are listed in multiplex PCR groups.

| **Locus** | **Forward Primer** | **Reverse Primer** |
| --- | --- | --- |
| **Group 1** |  |  |
| **G1D** | GATCTGTGGGTTTATAGGTTACA | CTACTCTTCCTACTCTTTAAGAG |
| **G10H** | CTCTTGCCTTACTTACATGG | ATCAGAGACCACCAAGTAGG |
| **G10J** | GATCAGATATTTTCAGCTTT | AACCCCTCACACTCCACTTC |
| **G10M** | TTCCCCTCATCGTAGGTTGTA | AATAATTTAAGTGCATCCCAGG |
| **G10X** | CCCTGGTAACCACAAATCTCT | TCAGTTATCTGTGAAATCAAAA |
| **Group 2** |  |  |
| **G1A** | TCCAGTGTCCTCCCTTTCT | AGATTAGTGAAAAAGAAGCAGG |
| **G10B** | GCCTTTTAATGTTCTGTTGAATTTG | GACAAATCACAGAAACCTCCATCC |
| **SRY** | GAACGCATTCTTGGTGTGGTC | TGATCTCTGAGTTTTGCATTTG |
